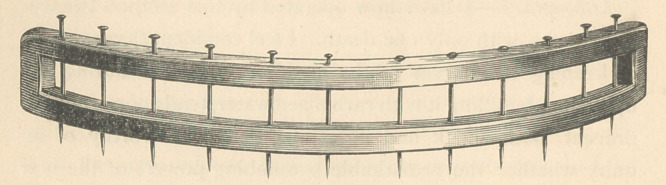# Recent Cases in Surgical Practice

**Published:** 1884-12

**Authors:** Edmund Andrews

**Affiliations:** Professor of Clinical Surgery. Chicago Medical College, Medical Department, Northwestern University; No. 6, Sixteenth Street, Chicago


THE CHICAGO
### Medical Journal and Examiner.

Vol. XLIX. DECEMBER, 1884. No. 6.

### Original Communications.

### Article I.

Recent Cases in Surgical Practice. *By* Edmund Andrews, m. d., ll.d., *Professor of Clinical Surgery, Chicago Medical College, Medical Department, Northwestern University. Read Before the Chicago Medical Society, November 10, 1884.*

The following cases are of interest because of the numerous unsettled questions which cluster around the operations referred to:

*Case 1. Gastrostomy.*—I may say, by way of preface, that this barbarous word is an etymological blunder, so far as literary usages are concerned. It is considered bad scholarship to compound a new term partly of Greek and partly of Latin. The author of this philological monstrosity seems to have attempted to enlarge the word gastrotomy, which literally signifies stomach-cutting, by the addition of a syllable to signify a mouth, so as to make the compound mean a cutting of the stomach to make a mouth—literally a stomach-mouth-cutting.

Now, the syllables gastro, from the Greek *gaster*, signify stomach, and the termination, tomy, is from the Greek *tome*, signifying an incision; but the intermediate syllable, *os*, or mouth, is Latin, which mixture of languages is condemned by all scholars. The author of the word would perhaps say that *stoma* in Greek is mouth, and therefore gastro-stoma would signify stomach-mouth. This, also, is an error. *Stoma* in composition, always resumes the latent found in the oblique cases, so that the word should then be gastrostomaty, and even then there would be nothing left in the compound to signify the cutting. To express the full meaning of stomach-mouth-cutting would require the form *gastro-stomatomy*, a term whose length and harshness of sound is sufficient for its condemnation. After all, it is not necessary in naming an operation to introduce a syllable merely to express the motive of the operator in performing it. Let us go back to the well-known term gastrotomy, or else say simply the making of a gastric fistula; for, after all, a mere hole in the stomach is by no means a mouth, and ought not to be called one. Above all things, if we must air our learning by manufacturing Greek technical terms, let us see to it that they are scholarly in composition and euphonious in sound, two important requisites which have been constantly ignored by a class of pedantic surgical authors.

Case I was a child aged 6 years. She swallowed some concentrated lye some months before I saw her, cauterizing the lower part of the œsophugus, and gradually inducing a stricture. Dr. E. P. Cook, of Mendota, an eminent and well-known surgeon, dilated the stricture, and sent the child home greatly improved, but after leaving his care she relapsed, and he sent her to me. When she arrived she had been unable to swallow anything for a number of days. I tried dilatation, and she improved for a time, regaining a partial power of swallowing liquid food. However, I found that I could not maintain the advantage gained, and after faithful trial it became evident that the patient was gradually starving. I therefore performed the so-called gastrostomy, or gastrotomy, if you prefer a scholarly term, in a room which had been sprayed for an hour with carbolic acid, but did not allow the spray to touch the peritoneum. Making the usual incision in the hypochondrium from near the xiphoid cartilage downward to the patient's left. I found the colon partly in the way, but pushing it downward, I drew out the stomach with long-toothed forceps, identified the viscus by the relations of the gastro-epiploic vein, and secured it to the abdominal wall by a long suture on each side. As I did not dare to let starvation go on several days longer, as recommended by some, I opened the stomach at once, and sewed the edges closely to the skin all around the incision. There was a good deal of shock, but reaction occurred. Union by first intention took place without difficulty and with no peritonitis. Peptonized food was regularly inserted, and on inspection, found perfectly digested except when meat was used. This whether raw or cooked, would be ejected from the wound unchanged, even when retained twenty-four hours. For some days the patient improved, but it soon became evident that most of the food, though digested, did not pass through the pylorus. It seemed that this orifice of the stomach required a little pressure to unfold it, and that whenever the stomach contracted for the purpose, the chyme escaped by the fistula into the dressings, and did not pass on into the intestines. A rubber pad, tight enough to stop the outflow, could not be tolerated. Dr. Wyllys Andrews therefore constructed a soft rubber valve, as follows: a rubber tube, having a thick circular flange at one end, an inch and a quarter in diameter, was provided with another similar rubber disk, perforated in the center, so that it could be slipped on over the tube. The terminal flange was now rolled up and inserted into the stomach, where it expanded into its disc-form again, the rest of the tube, of course, remaining outside. The perforated disk was then slipped on over the tube, and pushed down against the abdomen, pulling the inner disk well out against the inner wall of the stomach. Then, by clamping the tube outside of the outer disk, a perfect valve was obtained, which prevented leakage. I feared at first the effect of the inner disk on the walls of the stomach, but it produced no perceptible irritation. I purposely made the opening in the stomach pretty large, hoping at a future time to introduce my finger upward into the lower end of the œsophagus, and then by pushing down upon it a puncturing instrument with a guarded point, to restore the natural passage. The valve retained the food perfectly, and the patient took an abundance, and even learned to know when she was hungry and to call for her meals; but after a temporary improvement, she began slowly to fail, without obvious cause. The power of assimilation seemed gone, and she constantly grew weaker. By the thirty-fifth day after the operation it became evident that she was about at the end of her life. The last twenty-four hours were accompanied with an obscure fever.

*Case 2.*—An adult male patient. Six months ago, he swallowed some caustic ammonia, producing a contracting ulcer of the lower part of the œsophagus. In September last, he came to me, having been unable to swallow anything for some days. By a diligent use of bougies I dilated the stricture and restored the power to swallow liquid food. He then returned home, having learned the art of passing the bougie himself, and taking two instruments with him. However, he lost the art in some way, and in October returned again in the same condition as before. I tried diligently to dilate the stricture a second time, but could gain nothing except a slight temporary power of passing small quantities of liquids a day or two at a time. After a week or two I perceived that his progress was downward, and that he was slowly starving. Moreover, the points of the bougies seemed to be creating a local inflammation in the right lung, as if they were making a false passage in that direction. I therefore desisted from their use, and determined to operate early and not allow the patient to become as weak as the previous one. The operation was the same as in the former case, except that the opening was smaller. The same difficulty of regurgitation of the food occurred and was controlled by a similar valve. The patient is now doing well, and bids fair to recover. It is now thirty-two days since the operation, and there has been no sign of peritonitis. The plan pursued by Mr. Howse, of London, is two insert two rows of sutures in a peculiar manner to hold the stomach. Both he and many other surgeons advise to attach the stomach to the wound first, and to postpone the actual opening of that viscus until some five days later, and then make only a small orifice. This has the advantages claimed for it, doubtless, but is subject to the obvious objection that the surgeon must operate on patients who have already advanced so far towards fatal starvation that five days more of it, will, in spite of nutrient enemata, turn the scale to the side of death. I think that the best plan, on the whole, would be to make the mechanical parts of the operation something after Howse's plan, which is a very careful one, and then at the same operation insert a moderate meal of milk and raw egg, through a small aspirator tube. This will give the patient something to live on, and if needful could be repeated once a day, by holding a point of the stomach wall in such a manner that the insertion of the aspirator needle would not drag upon the stitches.

The statistics of this operation are not very cheerful. Of two hundred and seven cases recorded, forty were for cicatricial structure of the œsophagus, like those just detailed. Of these forty, twenty-one died, a mortality of more than half the patients; still, when they have no other hope of life, an operation which gives them one chance in two is a great benefit.

*Excision of the Rectum.*—I have at present two of these cases under treatment. Case first was epithelioma, not reaching down to the verge of the anus. I therefore saved the entire external sphincter. I have carried an incision from an inch in front of the anus back to the coccyx, opening the anus antero-posteriorly and cutting off the gut just above the verge. After dissecting it upward a little and tying numerous vessels, I separated the rectum from the pelvic chamber mainly with the finger, and divided the tube about three inches upward, just above the top of the cancer. There was no shock, and not much subsequent pain or inflammation. The second case was almost exactly a repetition of the first, except that the verge of the anus was involved in the disease, and consequently was dissected out with the rest. Both patients are doing well, and exceedingly comforatable, but it is too early to say whether there will be any return of the disease. Both of them should have applied for an operation at an earlier day. The statistics are much like those of epithelioma elsewhere. Billroth, of Vienna, thinks he permanently saves about one-third of the cases. Dr. Kelsey, in the *N. Y. Medical Record,* collected accounts of 608 cases, of which 140 died of the operation. Of those who survived the operation, one hundred were traced up. Thirty-one of the hundred were doing well at the end of one year, and seventeen were alive and well, with no return, at the end of three years.

*Lithoaplaxy.*—I have now operated by this method twenty-three times, with only one death. I feel confident that my plan of keeping the nerves of the bladder benumbed during the operation, by filling it with carbolized water, tends powerfully to prevent both shock and inflammation. It is worthy of inquiry whether the remarkable benumbing powers of the new agent, cocaine, would not enable us to operate both without ether and without shock.

*New Instrument for Operating on Varicocele.*—As the operations for destroying the veins in varicocele have produced occasional deaths, and in other cases ended in neuralgic scrotums, with or without atrophy of the testicle, many surgeons have followed the lead of Prof. Frank Hamilton, of New York, in preferring Sir Astley Cooper's plan of shortening the scrotum sufficiently to make it its own suspensory bag. Formerly this operation was vexatious, because the imperfect character of the old-fashioned adhesive plasters rendered it difficult to support the sutures sufficiently to secure a union by first intention. Now that we have the rubber plaster, which never lets go its grip, we can cut the scrotum very short, and still hold the wound firmly together, and secure a triumphant success.

Sundry clamps have been invented to hold the skin of the scrotum firmly while the surgeon cut it off and sewed it up. The evil of the clamp is that it compresses the arteries, so that after cutting away the pouch the surgeon is unable to find and ligate the vessels. If he sews up the wound without attending to this point, experience shows that after the clamp is removed, hemorrhage often takes place inside the scrotum, distending it with clot, and forcing open the wound, thus delaying the cure. To meet this difficulty, I have devised a kind of varicocele bow, which I here present. It consists of two curved steel parallel bars, connected at the ends and enclosing a slot three-eighths of an inch wide between them. Twelve holes are drilled through the bars of a size to admit ordinary pins. The surgeon draws the scrotum through the slot to such a distance as he deems sufficient, and secures it there by inserting, one by one, as many pins as he finds necessary to hold the pouch securely. He then cuts off the scrotum outside the convex border of the bars. As the scrotum is not pinched by the instrument, the blood spouts freely, especially from the artery at the raphe, and the operator can carefully and deliberately secure every bleeding point. This being accomplished, he sews the cut edges together, and then drawing out the pins removes the bow and applies his plasters. The neatness and dispatch of the operation are thus greatly enhanced.

No. 6, Sixteenth Street, Chicago.

## Figures and Tables

**Figure f1:**